# Modelling a new approach for radio-ablation after resection of breast ductal carcinoma in-situ based on the BAT-90 medical device

**DOI:** 10.1038/s41598-021-03807-6

**Published:** 2022-01-07

**Authors:** Anna Sarnelli, Matteo Negrini, Emilio Mezzenga, Giacomo Feliciani, Marco D’Arienzo, Antonino Amato, Giovanni Paganelli

**Affiliations:** 1 Medical Physics Unit, IRCCS Istituto Romagnolo per lo Studio dei Tumori (IRST) “Dino Amadori”, Via P. Maroncelli 40, 47014 Meldola, FC Italy; 2 Istituto Nazionale di Fisica Nucleare, Sezione di Bologna, 40126 Bologna, Italy; 3 Medical Physics Unit, ASL Roma 6, Via Borgo Garibaldi 12, 00041 Albano Laziale, RM Italy; 4 BetaGlue Technologies Spa, Lungadige Galtarossa 21, 37133 Verona, Italy; 5 Nuclear Medicine Unit, IRCCS Istituto Romagnolo per lo Studio dei Tumori (IRST), Dino Amadori”, Via P. Maroncelli 40, 47014 Meldola, FC Italy

**Keywords:** Physics, Cancer models

## Abstract

The majority of local recurrences, after conservative surgery of breast cancer, occurs in the same anatomical area where the tumour was originally located. For the treatment of ductal carcinoma in situ (DCIS), a new medical device, named BAT-90, (BetaGlue Technologies SpA) has been proposed. BAT-90 is based on the administration of ^90^Y β-emitting microspheres, embedded in a bio-compatible matrix. In this work, the Geant4 simulation toolkit is used to simulate BAT-90 as a homogenous cylindrical ^90^Y layer placed in the middle of a bulk material. The activity needed to deliver a 20 Gy isodose at a given distance z from the BAT-90 layer is calculated for different device thicknesses, tumour bed sizes and for water and adipose bulk materials. A radiobiological analysis has been performed using both the Poisson and logistic Tumour Control Probability (TCP) models. A range of radiobiological parameters (α and β), target sizes, and densities of tumour cells were considered. Increasing α values, TCP increases too, while, for a fixed α value, TCP decreases as a function of clonogenic cell density. The models predict very solid results in case of limited tumour burden while the activity/dose ratio could be further optimized in case of larger tumour beds.

## Introduction

The widespread use of screening mammography allows the early detection of small size unifocal Ductal Carcinoma in Situ (DCIS) that represents up to 25% of all new detected breast cancers^1–3^.

To date, the optimum treatment for patients with DCIS is still an open question. In particular, the role of Radiotherapy (RT) after Breast Conserving Surgery (BCS) to control microscopic residual disease and, then, to prevent local recurrence (LR), is a matter of debate. Different studies report that at least 75% of LR involves the lumpectomy site or the index quadrant, raising concerns that in the periphery of the cavity, after an incomplete eradication, there are still residual cancer cells that are not visible to standard imaging modalities^4^.

The use of RT should be proposed selectively to patients at high risk of LR or progression to invasive breast cancer, especially because of its long-term side effects, such as cardiotoxicity^5^. In the past decades, several strategies have been proposed to perform a risk stratification of DCIS patients with respect to a number of known prognostic parameters, such as age, tumour size and nuclear grade^6–8^. In fact, a risk-based approach could improve the ability to select patients that should experience a real benefit from RT, in comparison to the patients that could be over-treated with it. The impact of RT after BCS in DCIS patients has been deeply investigated in Amichetti et al.^7^ and Cutuli et al.^9^ and references therein. These studies’ results provide evidence that, despite the efforts to identify a low-risk group that could undergo BCS alone, RT reduces the relative risk for ipsilateral tumor recurrence by almost 50% in all subgroups, except for younger patients where the relative efficacy of RT was less evident^5,10^.

In this context, new irradiation modalities and schedules, such as Accelerated Partial Breast Irradiation (APBI), represent emerging therapeutic options that combine the benefits of a localized irradiation of the tumour bed after BCS with a reduced treatment time. APBI includes several irradiation modalities: brachytherapy (intracavitary and interstitial^11,12^), external beam radiation therapy (EBRT^13,14^), intraoperative radiation therapy (IORT^15^) and proton therapy^16^.

In 2017 an updated consensus statement about APBI was published by ASTRO/SSO that reports revised inclusion/exclusion criteria of the “*suitable*” and “*cautionary* “patients to be treated by Partial Breast Irradiation (PBI), including pure DCIS^17^. The “*suitable*” DCIS patient group includes patients with low-risk DCIS as per RTOG 9804 criteria^18^, which are: age ≥ 50 y.o., screen-detected lesions with low to intermediate nuclear grade, size < 2.5 cm and resected margins negative at ≥ 3 mm.

In the context of PBI modalities, BetaGlue Technologies SpA has proposed a new approach for the irradiation of surgical margins following resection of DCIS with BCS. In fact, BetaGlue Technologies SpA has developed a new compound (BAT-90) based on ^90^Y β-emitting microspheres, embedded in a bio-compatible matrix and delivered to target with a proprietary delivery system. The background of such an approach is based on the long-term clinical experience developed in the field of Selective Internal Radiotherapy (SIRT), also called Transarterial Radioembolization (TARE), whereby the arterial infusion of ^90^Y-coated microspheres is performed into liver segments harbouring primary or secondary liver tumors^19^.

BAT-90 is composed of two individual components, categorized as medical devices themselves (both already CE-marked and FDA-approved): a surgical sealant matrix and ^90^Y-coated resin microspheres. Their mixture, which is performed in an accredited hospital radio-pharmacy, is administered onto the surgical bed following the resection of DCIS through a dual chamber syringe, under visual guidance. The adhesive begins to polymerize within 20–30 s. and reaches maximum bonding strength in 2 min. Once injected, BAT-90 remains adherent to the resection bed, delivering the ^90^Y dose selected to eradicate the minimal residual disease potentially left behind by the surgeon.

According to the data reported in the literature, an absorbed dose of 20 Gy (> 18 Gy) is deemed sufficient to control local relapse^8,15,20^.

Preliminary in-vitro and in-vivo tests have been performed. In the former ones, PET/CT images demonstrated that there were neither dissociations of BAT-90 nor alterations in its distribution on the injection site. The latter ones were conducted in rodent models (mouse and rabbit), as well as in a large mammal (pig) model; they confirmed that BAT-90 can be applied as filling material for a surgical cavity and can provide a locoregional treatment of residual tumour cells potentially present along its margins (BetaGlue Technologies, data on file).

The aim of this study was to calculate the ^90^Y activity corresponding to a given radio-ablative dose to the surgical bed, as a function of tumour bed size and tumour volume coverage required. A Monte Carlo simulation of a homogenous water or adipose tissue phantom was implemented using the Geant4 toolkit to evaluate the absorbed dose in tissue layers adjacent to the tumour bed.

Event by event Monte Carlo simulations are powerful tools to investigate the interaction of ionizing radiation in media. In particular, Geant4 is a Monte Carlo toolkit widely used in medical physics with applications in dosimetry, micro- and nano- dosimetry, imaging, radiation protection and nuclear medicine. Nowadays, Geant4 offers physics models to describe interaction of low energy electrons that are particularly well suited for medical applications^21^, and tools for the benchmarking of such applications^22^. The toolkit also includes a package to model nuclear decays^23^ that are of interest for medical applications, such as the ^90^Y used in this study.

In the present study, the results of the Monte Carlo simulation are then used to perform a radiobiological analysis of the treatment efficacy, considering different clinical and practical settings.

## Results

The α/β value for breast tumour was assumed equal to 3.6 Gy^24^ while α assumed a wide range of values (0.02–0.90 Gy^−1^), in order to account for the heterogeneity of patient radio-sentivity. β values varied too according to the relationship: β = α/(α/β). The assumed sublethal damage repair constant is μ = 0.5 h^−1^^25^.

For the residual tumour cell density, the following scenarios were considered: (*a*) constant density equal to 10^4^, 10^5^, 10^6^, 10^7^ cells/cm^3^; (*b*) linearly decreasing density with ρ_0_ equal to 10^4^, 10^5^, 10^6^, 10^7^ cells/cm^3^.

For the range of α-values considered and two density models, the resultant SF, EUD_2Gy_ and TCP values were estimated.

**Figure 1:**
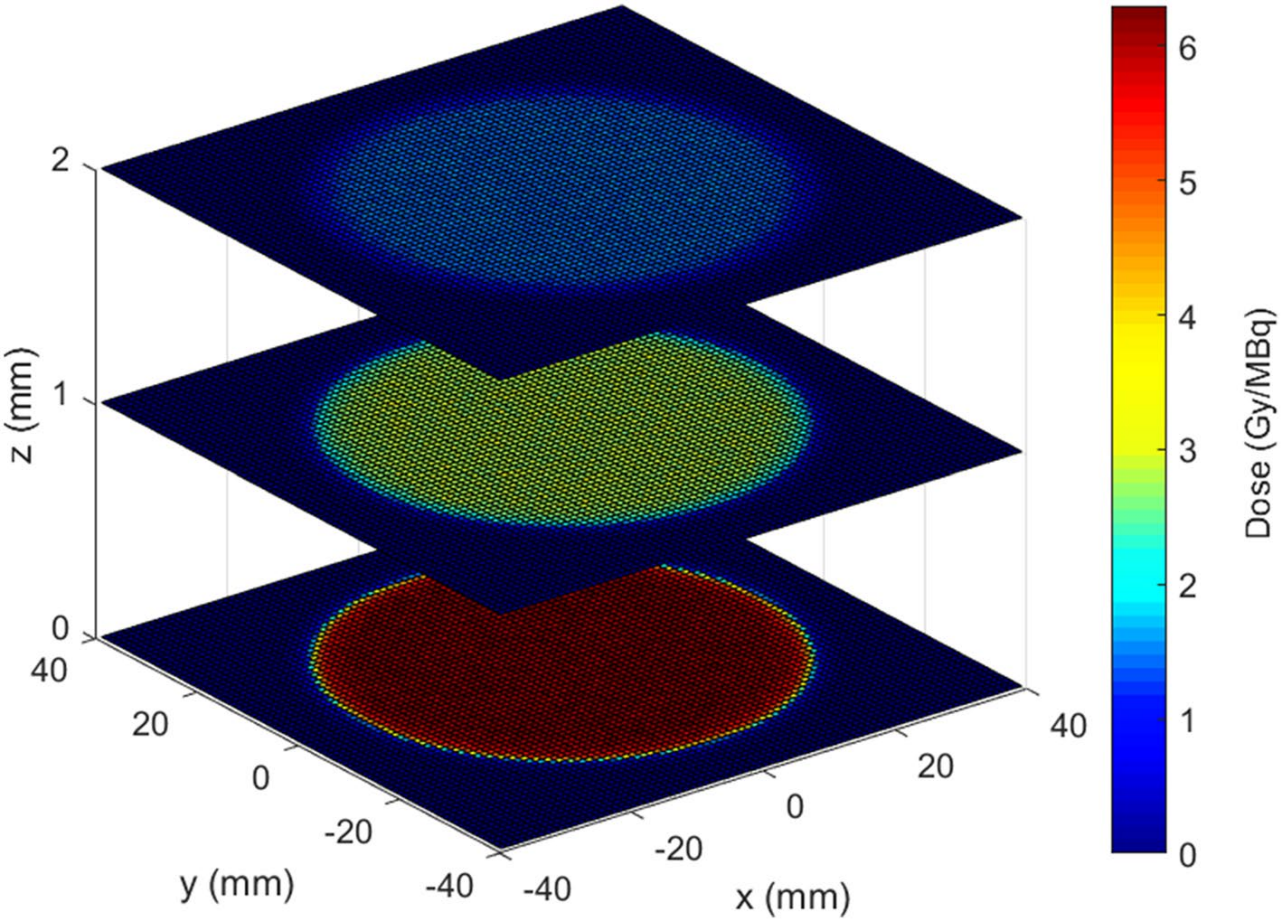
Orthogonal view of the dose distribution per unit of activity (Gy/MBq) on three parallel planes: the tumour bed (z = 0 mm) and at 1 and 2 mm distance. The values are obtained for circular tumour bed with R = 30 mm and for a BAT-90 thickness t = 0 mm.

**Figure 2:**
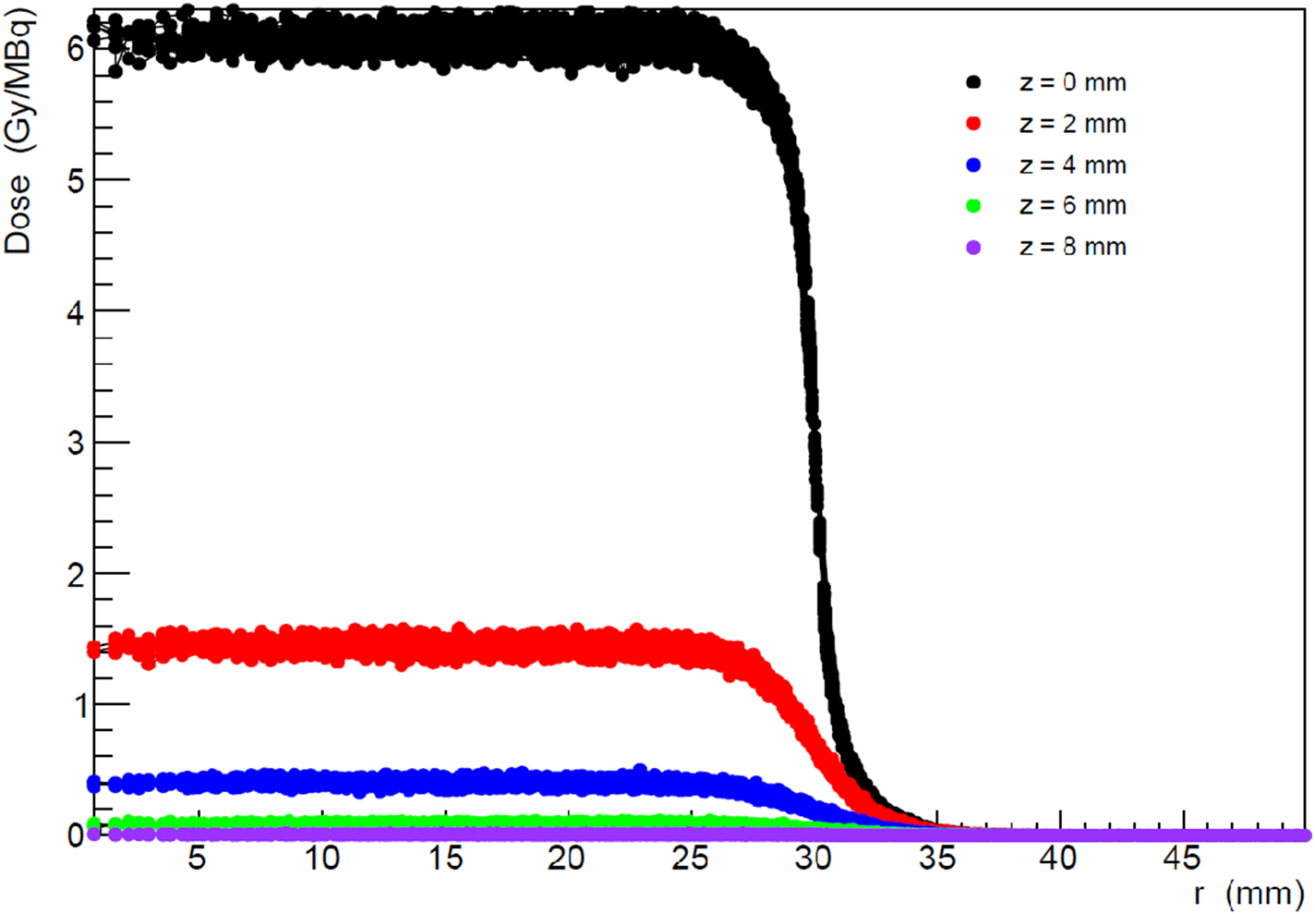
Dose values per unit of activity (Gy/MBq) in each voxel located on planes at fixed distances from the tumour bed (colour code) as a function of the radial distance r from the center of the treated region. The values are obtained for a circular tumour bed with R = 30 mm and for a BAT-90 thickness t = 0 mm. The plot illustrates the steep fall of the dose when moving away from the tumour bed, both in z and in r, and the homogeneity of the dose values obtained in the tissue adjacent to the treated region.

Figure 1 shows a pictorial representation of the dose distribution at different distances z from the tumour bed plane, corresponding to a circular tumour bed with R = 30 mm and negligible BAT-90 thickness (t = 0 mm). For the same configuration, Fig. 2 shows all dose values as a function of the radial distance r from the center of the tumour bed, for all pixels located on planes at various distances from the tumour bed plane. For R = 30 mm, in each plane at constant z, that means parallel to the tumour bed plane, the dose delivered by ^90^Y is uniform for r < 25 mm and steeply decreases when r > 25 mm, in correspondence with the edge of the tumour bed. Along the same lines, the absorbed dose becomes negligible at about z ≈ 10 mm from the tumour bed, according to the maximum range of ^90^Y β-particles that is 11 mm in water.

**Figure 3:**
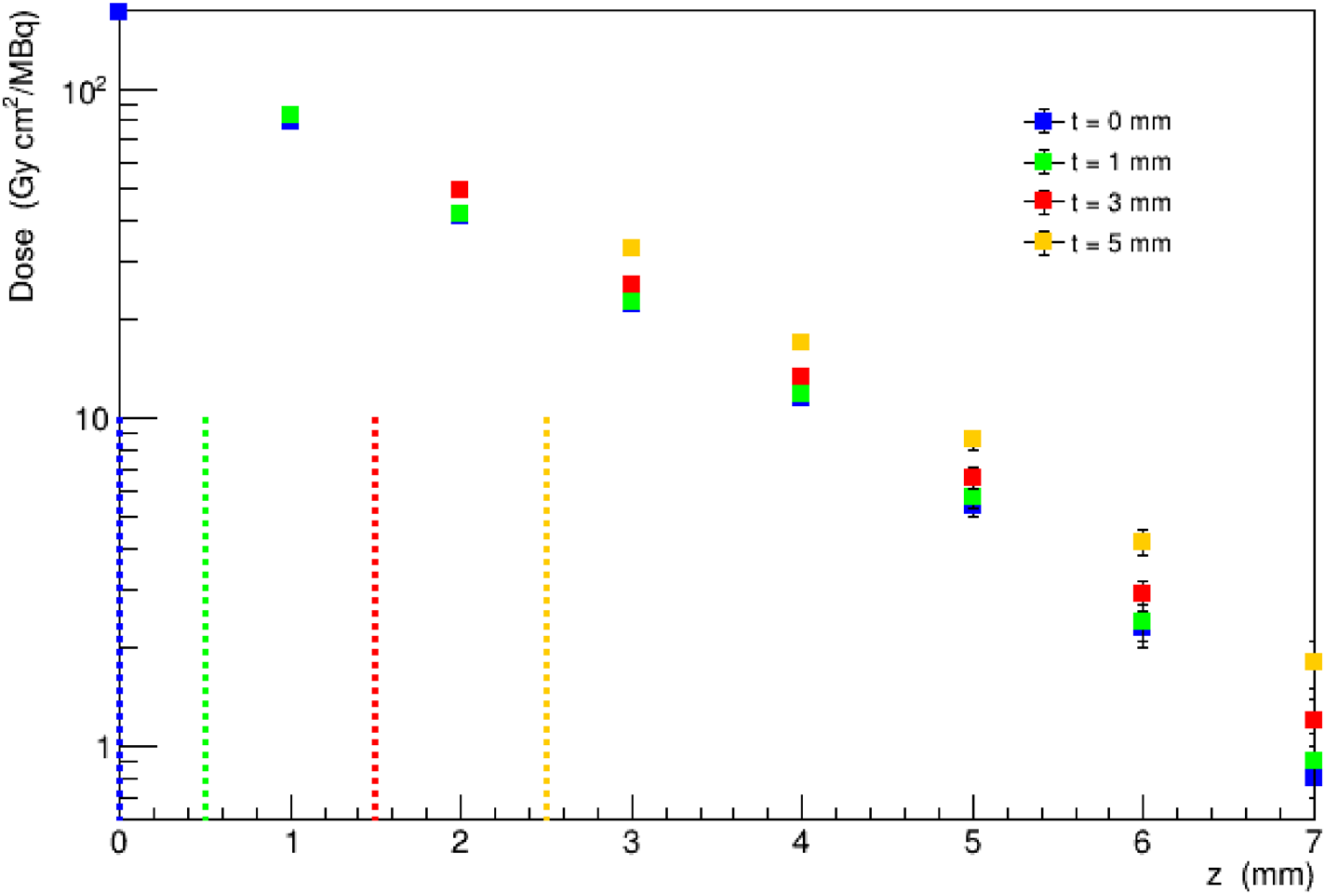
Dose per activity on unit area values in the water bulk material, on planes located at different distances z from the tumour bed, for different thicknesses t of the BAT-90 layer (colour code). The vertical dashed lines correspond to the upper surface position of the BAT-90 layer (corresponding to t/2, since the layer is centered in the coordinate system). Dose values are reported only for the bulk material outside BAT-90 (for z > t/2).

Figure 3 shows the absorbed dose as a function of the distance z from the tumour bed plane, comparing the results obtained for thicknesses of the BAT-90 layer in the range 0 mm < t < 5 mm. The results are expressed in terms of absorbed dose (Gy) per unit of activity concentration (MBq/cm^2^). In the calculation, water is assumed for both the bulk material and the BAT-90 composition. The simulation setup is symmetric around the center of the coordinate system; therefore, for a given thickness t, the cylinder represents the radioactive material covering the region z ≤|t/2|. For this reason, to help in the interpretation of the results, the upper surface position of the BAT-90 material is indicated as a vertical dashed line in correspondence with + t/2 in the plot in Fig. 3. In particular, the doses at z ≤|t/2| should be considered as doses delivered within the BAT-90 layer itself and are not shown in the figure, while the doses at z >|t/2| are the doses delivered to the surrounding tissues (therapeutic dose). It can be observed that the very large doses observed on the tumour bed plane obtained for t = 0 are caused by the high concentration of radioactive nuclei on the plane itself. It can also be observed that, except for the region very close to the treatment plane for the t = 0 mm case, the dose profile to the tissues surrounding the treated region shows just a mild variation with the simulated thicknesses. These values are presented in Table 1, which also reports the activity (normalized for the tumour bed surface) to be administered to deliver a 20 Gy isodose at different distances from the middle plane of the BAT-90 layer.

**Table 1:** Activity per unit area and dose per activity on unit area of the tumour bed in a water bulk material in the points facing the treated region, at different distances z from the central plane of the BAT-90 layer.

t (mm)	z = 0 mm			z = 2 mm			z = 4 mm			z = 6 mm			z = 8 mm		
	k (mm)	Activity (MBq/cm^2^)	Dose (Gy cm^2^/MBq)	k (mm)	Activity (MBq/cm^2^)	Dose (Gy cm^2^/MBq)	k (mm)	Activity (MBq/cm^2^)	Dose (Gy cm^2^/MBq)	k (mm)	Activity (MBq/cm^2^)	Dose (Gy cm^2^/MBq)	k (mm)	Activity (MBq/cm^2^)	Dose (Gy cm^2^/MBq)
0	0	0.11	172.0	2	0.49	40.9	4	1.8	11.4	6	8.7	2.3	8	100	0.2
1	–	–	–	1.5	0.48	41.6	3.5	1.7	11.7	5.5	8.3	2.4	7.5	67	0.3
3	–	–	–	0.5	0.41	49.0	2.5	1.5	13.3	4.5	6.7	3.0	6.5	50	0.4
5	–	–	–	–	–	–	1.5	1.2	16.8	3.5	4.8	4.2	5.5	28	0.7

Values of t in the range 0–5 mm are considered. k is the thickness of the tissue shell irradiated. Only values in the bulk material outside the BAT-90 layer (z > t/2) are reported.

**Figure 4:**
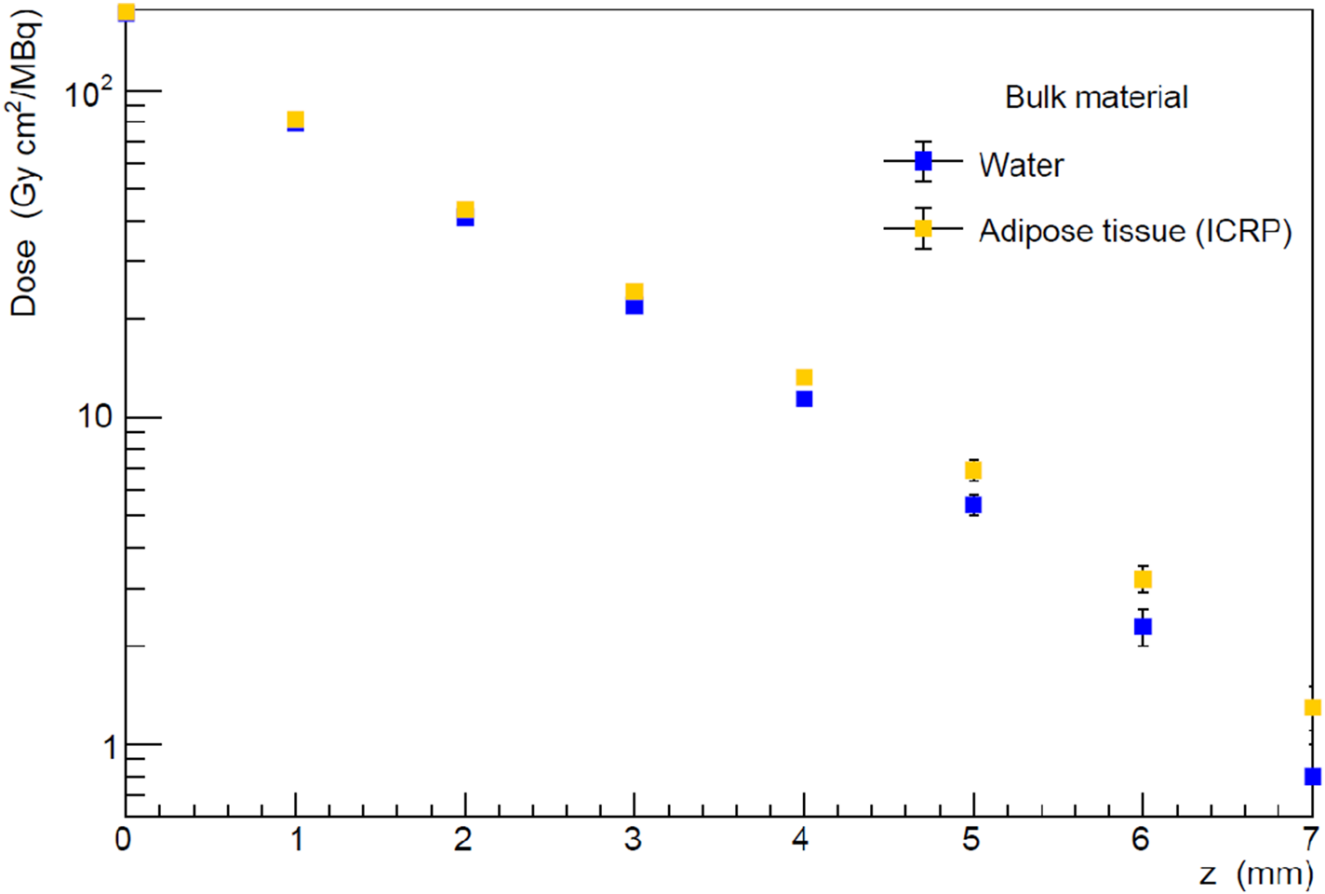
Dose per activity on unit area values for water and adipose tissue bulk material on planes at different distances z from the tumour bed. The values are obtained for a BAT-90 thickness t = 0 mm. The dose in adipose tissue is larger than the one in water and the effect increases with z.

Figure 4 shows a comparison of the results obtained using adipose tissue instead of water as a bulk material, for t = 0 mm. The electrons have the same mass-density range in adipose tissue and in water. However, because of the different density, the dose profile in adipose tissue is shifted along the z axis with respect to the dose profile in water by a factor 1.053, i.e. the ratio between the water and the adipose tissue densities. As a consequence, the 20 Gy isodose is reached at a greater depth in the adipose tissue than in water.

Table 2 reports the coefficients of the best fit curves for a normalized dose d profile as a function of the z distance, for different t. The a1 coefficients (Gy cm^2^/MBq) decreases as the BAT-90 layer increases, while the a2 coefficients are almost independent from the t parameter. Most likely, this is because for z ≤|t/2| almost all ^90^Y β-particles energy is delivered to the BAT-90 layer, with negligible dose contribution to tissues.

**Table 2:** Coefficients of the best fit curves, with 95% confidence interval (round brackets), to the normalized dose profile as a function of the z distance and the t thickness.

t (mm)	z_0_ (mm)	a1 (Gy cm^2^/MBq)	a2 (mm^−1^)
0	0	170.9 (166.1, 175.7)	− 7.2 (− 7.6, 6.8)
1	0.5	115.7 (112.5, 118.9)	− 6.7 (− 6.9, -6.4)
3	1.5	68.5 (66.3, 70.8)	− 6.7 (− 7.0, -6.4)
5	2.5	46.3 (46.3, 47.83)	− 6.8 (− 7.11, -6.5)

**Figure 5:**
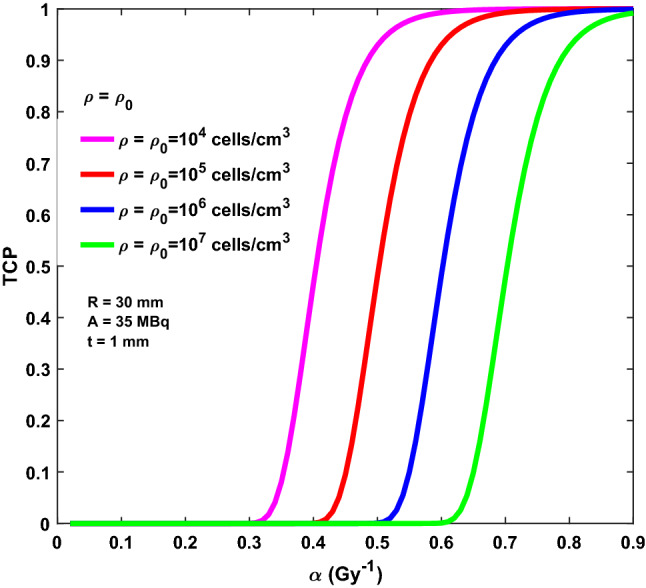
TCP as a function of the α values for R = 30 m, A = 35 MBq and BAT-90 layer t = 1.0 mm. Different constant residual clonogenic cells density are considered: 10^4^ cells/cm^3^ (magenta line); 10^5^ cells/cm^3^ (red line); 10^6^ cells/cm^3^ (blue line); 10^7^ cells/cm^3^ (green line).

**Figure 6:**
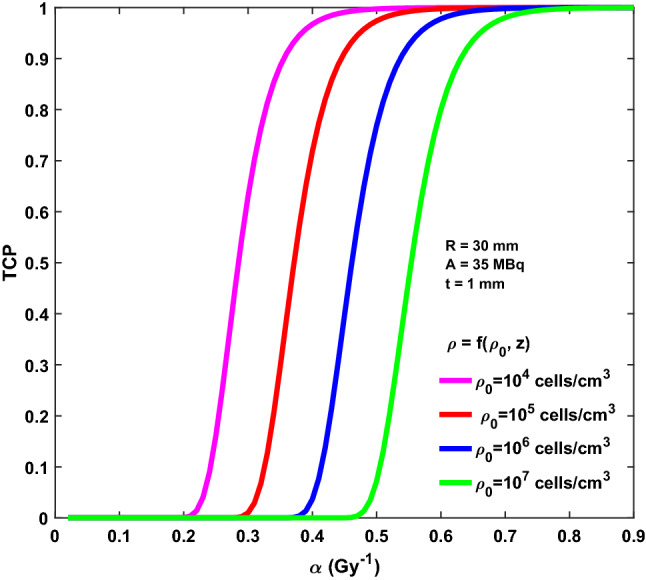
TCP as a function of the α values for R = 30 mm, A = 35 MBq and BAT-90 layer t = 1.0 mm. A linearly decreasing residual clonogenic cells density is considered with different ρ_0_: 10^4^ cells/cm^3^ (magenta line); 10^5^ cells/cm^3^ (red line); 10^6^ cells/cm^3^ (blue line); 10^7^ cells/cm^3^ (green line).

Finally, Figs. 5 and 6 report TCP based on the Poisson model as a function of the α values for different clinical settings. As an example, a tumour bed with R = 30 mm is considered, for which an activity A = 35 MBq is needed to obtain a 20 Gy isodose, assuming t = 1.0 mm and k = 3 mm. Two different clinical scenarios are considered, corresponding to a constant residual clonogenic cell density (Fig. 5) and a linearly decreasing residual clonogenic cell density (Fig. 6), respectively. In the former case, four values of cell density are assumed ranging from 10^4^ to 10^7^ cells/cm^3^. For the latter case, the cell density is considered to be decreasing linearly as a function of the z distance from the BAT-90 layer, with a maximum value ρ_0_ at z = z_0_ and reaching null density at z = z_0_ + k where the isodose of 20 Gy is reached. Also in this case the same values of ρ_0_ considered in Fig. 5 are used for calculation.

As a general rule, for increasing α values (i.e., decreasing radio-resistance), TCP increases as well. As expected, for a fixed α value, TCP increases as ρ (ρ_0_) decreases. Moreover, the TCP decreases as the size of the tumour bed increases (R) (Supplementary Fig. S1), that is the residual disease is more likely to be eradicated in the smaller cavities. Most importantly, the thickness of the BAT-90 layer plays an important role on the tumour control, with TCP decreasing for increasing layer thickness, t (Supplementary Fig. S2).

**Figure 7:**
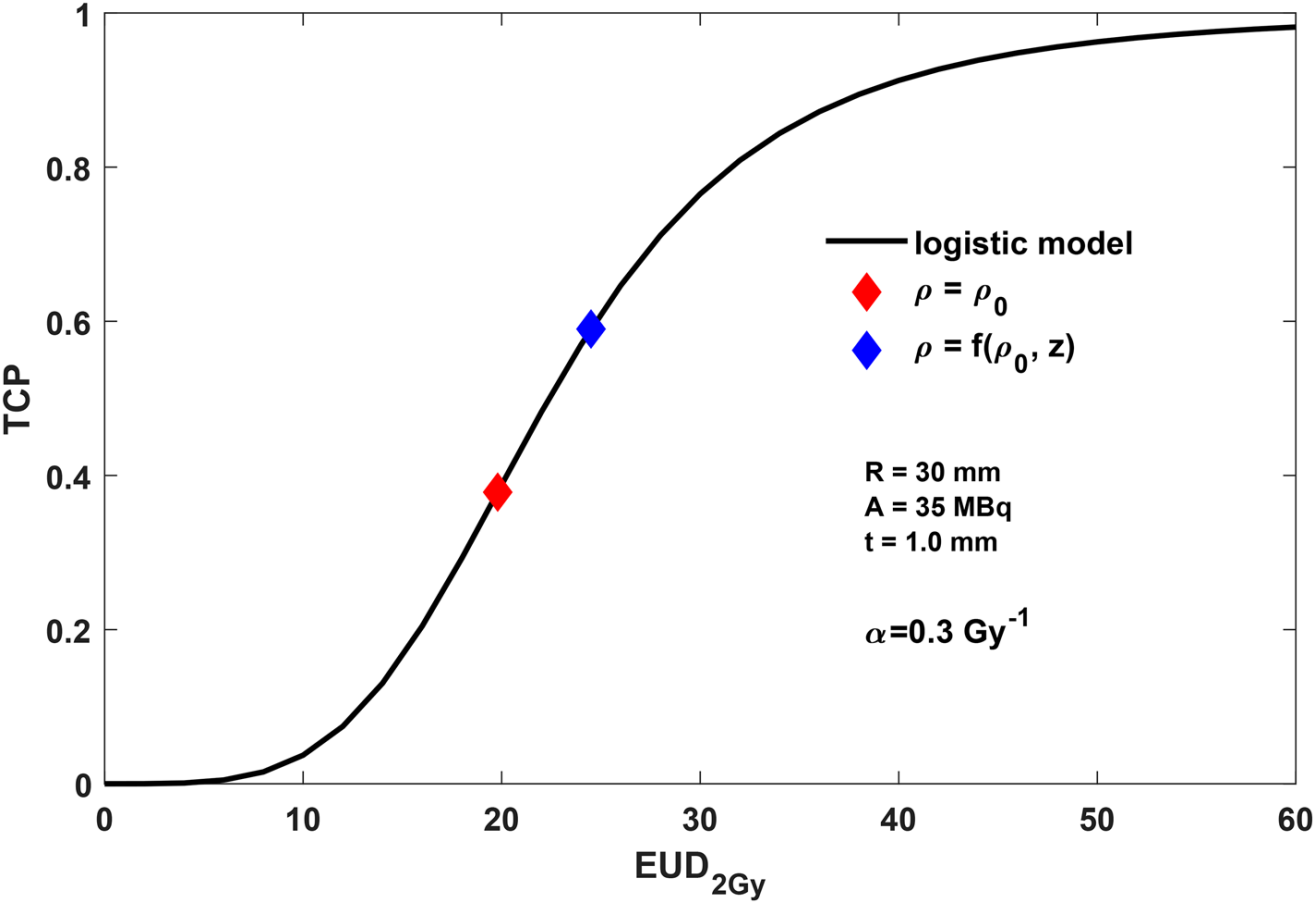
TCP based on logistic model (solid line) as a function of the EUD_2Gy_. Estimated TCP value for R = 30 mm, A = 35 MBq and BAT-90 layer t = 1.0 mm in case of constant ρ (red marker) and linearly decreasing ρ (blue marker). The α is equal to 0.3 Gy^−1^.

The plot in Fig. 7 reports the TCP logistic model (solid line) as a function of the EUD_2Gy_. The values assumed for D_50_ and γ were 22.39 Gy and 1.01, respectively, according to the analysis performed in Giraudo et al*.*^24^. For a tumour bed with R = 30 mm, A = 35 MBq, t = 1 mm and k = 3 mm, the SF values for a fixed α = 0.3 Gy^−1^ were derived for both constant and linearly decreasing residual clonogenic cell density, that correspond to EUD_2Gy_ = 19.8 Gy and EUD_2Gy_ = 24.5 Gy, respectively. The TCP value estimated is 0.39 in case of constant density (red marker) and 0.59 for linearly decreasing cell density (blue marker).

**Figure 8:**
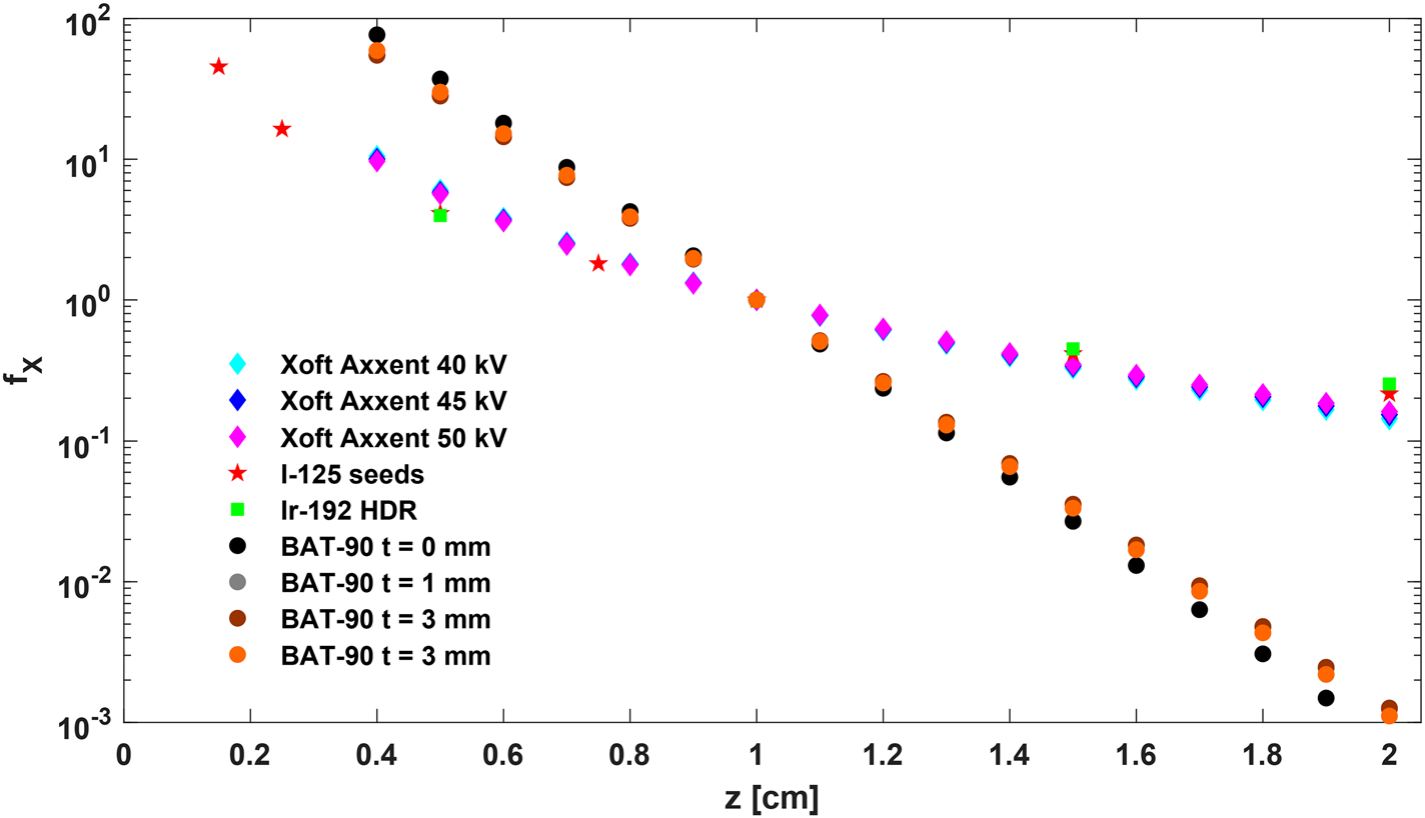
Dose fall-off f_X_ of the source X along the transversal axis. It includes the absorption, the scatter and the geometrical distribution of the radioactive material. The sources considered are Xoft Axxent at 40 kV, 45 kV and 50 kV (light blue, blue and magenta diamond), I-125 seeds implants (red pentagram), Ir-192 HDR (green square) and BAT-90 with t = 0 mm, t = 1 mm, t = 3 mm and t = 5 mm (black, grey, brown and orange circle).

At the end, the Fig. 8 reports the dose profiles for BAT-90, f_BAT-90_, for different layer thicknesses, and the other brachytherapy sources employed in clinical settings (electronic sources, I-125 seeds implants, Ir-192 HDR). For all t values, BAT-90 dose levels are higher in the near-tumour zone and the dose fall off is more rapid than the other sources. In fact, when the prescription point is fixed at 1 cm, f_BAT-90_ at 2 cm is 100 times smaller than f_X_ for any other brachytherapy approach.

## Discussion

The BAT-90 device may represent a promising approach in the context of APBI, whose main rationale is to offer an alternative option vs. whole breast irradiation (WBI) after lumpectomy in selected patients with early breast cancer, including DCIS. This is especially true in the light of past studies highlighting the effectiveness of molecular radiotherapy with ^90^Y as a radiotherapy boost in breast cancer^26,27^. Actually, BAT-90 offers the same potential benefits provided by the therapeutic options encompassed by IORT, without its cost, limitations and logistical complexity. In fact, as the RT is delivered immediately after surgical resection with BAT-90, while the patient is still in the operating room, the longer and quite often not being adhered to schedules associated with EBRT are overcome, with potential important benefits for patient’s management and clinical outcome. With BAT-90, the treatment time equals the time the device is in contact with the surgical bed. During this period, the device continuously delivers the planned dose, which patients receive without the need to comply with any scheduled visit to the RT facility.

If BAT-90 is compared to intracavitary brachytherapy (IB) with Ir-192, the dose is continuously delivered in both approaches. However, the brachytherapy schedule consists of 10 fractions delivered twice per day, making BAT-90 more practical and convenient for the patients. The advantage of BAT-90 is even more evident if this device is compared with the multi-catheter interstitial brachytherapy (MBI), the oldest and time-tested technique of breast brachytherapy. As for MIB, a dose of 34 Gy in 10 fractions is delivered over a period of 5–7 days, with 2 fractions delivered daily 6 h apart. Moreover, this therapeutic option is technically demanding, is not very popular and is almost replaced by the IB approach.

BAT-90 offers the relevant advantage to deliver a large dose in a continuous way to a limited portion of the breast tissue surrounding the resection cavity. Thanks to the finite range of beta particle penetration, an important absorbed dose drop-off is observed that allows sparing the surrounding normal tissues, which are instead potentially included even in very selective forms of EBRT, which is normally associated with a better outcome. Therefore, the device could play an important role in avoiding EBRT side effects, such as lung fibrosis or cardiotoxicity, which are especially relevant in DCIS patients with long life expectancy.

The advantage of rapid fall-off of the dose profile is also evident if comparing f_BAT-90_ with the same brachytherapy sources in both point-source approximation (Fig. 8) and line-source approximation. In fact, in this last case the advantage is even more evident as $G(r, {\theta }_{0})\approx {r}^{-1}$. Two main aspects are improved by BAT-90 approach with respect to the standard brachytherapy: the beta sources distributed in the planar geometry, in contact with the tumour bed, allow the deposition of a higher tumour killing dose in correspondence with the region of interest, while the short range of the particles guarantees the sparing of healthy tissues.

Table 1 reports the activity per unit area of the tumour bed to be injected with BAT-90 in order to obtain the therapeutic 20 Gy isodose at a given distance z from the tumour bed plane and also the corresponding dose per activity on unit area and activity of the tumour bed. These distances correspond to different tissue shell thicknesses k = z-z_0_ to be irradiated, depending on the device layer, t, given z_0_ = t/2. The activities corresponding to the doses inside the BAT-90 volume, i.e. z < t/2, are not reported. The data show the rapid dose fall-off as a function of z, as expected for beta particles. It can be observed that the values obtained in the region close to the tumour bed show just a mild dependence on BAT-90 thickness. Larger variations with t can be observed for larger distances (z ≅ 8 mm), where the dose is more than one order of magnitude smaller than that close to the tumour bed. In fact, at z = 8 mm the dose difference between the t = 1 mm and t = 3 mm configuration is approximately 30%. The activity to be injected can be obtained for any given z value from Eq. 2 and the coefficients in Table 2.

The radio-ablation of the surgical margins based on BAT-90 administration at the time of surgery could have an even greater effect thanks to its immediacy. In fact, according to the findings observed in Belletti et al.^28^, beside the well-known tumoricidal effect of radiations, a RT concurrent with surgery could alter the tumour microenvironment, enabling prompt suppression of cancer cell growth and migration, reducing the risk of local recurrence even further. Moreover, the immediate delivery of RT to the breast allows an accurate targeting, reducing the risk of a geographic miss.

It is worth noting that when comparing treatments delivered with different radiation quality, the ability to produce a biological damage per unit of delivered dose could be not the same. The radiation quality is characterized by the ionisation density which is quantified by the linear energy transfer (LET) while the concept of relative biological effectiveness (RBE) is therefore used to incorporate this effect in a radiobiological framework. Recent research estimated an RBE < 1 for IORT performed with high-energy electrons produced by linear accelerators, while an increased RBE is reported for low energy x-rays (LET ~ 3 keV/µm)^29^. Interestingly, the LET of beta particles emitted from ^90^Y is about ~ 1 keV/ μm, and about 5 times higher than LET value for 6 MV X-rays (~ 0.2 keV/μm^30^), i.e. the typical voltage used in the treatment of breast cancer with external beam radiation therapy. In the present study a tumoricidal endpoint of 20 Gy was considered, corresponding to an initial dose rate of about 0.2 Gy/hr. Of note, recent evidence reported the effectiveness of molecular radiotherapy performed with a high dose rate of ^90^Y (0.077–0.77 Gy/hr) in a number of cell lines irradiated in vitro^31^. Taken together, these results would seem to suggest that locoregional therapy with ^90^Y has the potential to contribute to the efficacy of intraoperative tumour-bed irradiation.

Other factors could play an important role in supporting the use of BAT-90 for radio-ablation purposes: (1) the medical device is placed directly onto the tumour bed and delivers a personalized dose immediately after the surgical excision, without issues of treatment compliance potentially associated with EBRT; (2) the optimal dose is thus delivered to the minimum required volume of the target tissues, sparing the surrounding tissues even more than EBRT; (3) the finite range of penetration of the beta particles offers very likely advantages in terms of cosmetic outcomes for this form of local RT, by maintaining a safe BAT-90 layer surface distance of at least 1.0 cm from the skin; considering that the average thickness of epidermis and dermis is about 1.0–1.5 cm in the breast area, BAT-90 is theoretically suited to be used also in more superficial surgical beds following breast-conserving surgery.

One of the possible limitations of this study is the assumption of a single sub-lethal damage repair constant, µ = 0.5 h^−1^, derived from previous literature findings based on a mono-exponential repair mechanism. This value has been used to successfully account for sub-lethal damage repair in targeted radionuclide therapy and in brachytherapy applications^32–34^. However, it is widely agreed that the sub-lethal damage repair constant may vary considerably with the dose rate and with the specific tumor cell line. Further to this, the mechanism of sub-lethal damage is not yet fully understood and different models have been developed to describe the repair mechanism (e.g. mono-exponential repair, multi-exponential repair and reciprocal time repair^34,35^. In a number of clinical scenarios there is no clear justification for preferring one model over another. In particular, multi-exponential repair models require knowledge of several parameter values, which sometimes hinder their clinical implementation. On the other hand, the reciprocal time repair model emulates the slowing down characteristics of multi-exponential models and requires just one parameter^34^.This model provided a good fit to a wide range of DNA repair data^35^. Assessing the impact of different repair half-times and models on the efficacy of radioablation using BAT-90 is beyond the scope of this paper but is certainly desirable for future work.

It is well known that a large number of factors will influence TCP in a real tumour, such as oxygenation status, tumour doubling time, dose-delivery pattern, tumor cell density and so on. Being aware that including all such factors in a single model is not feasible, in the present study we concentrated on the effect of cell density, ρ, directly related to the TCP through Eqs. (7) and (9).

The trend of TCP as a function of radio-resistance and the clonogenic cell density shows that the smaller the tumour bed, the higher is the probability to perform a curative treatment. However, these findings are strongly dependent on the assumptions made in the theoretical model. In particular, two clinical scenarios have been modelled, with constant and linearly decreasing residual clonogenic cells density, respectively, but no definitive ρ_0_ values are available in the literature. With increasing value of ρ_0_, the surviving clonogenic cells N_s_ increases as well and the TCP decreases for the planned dose. The same holds true for the radiobiological parameters employed in the TCP model. Even if there is a wide consensus about an α/β ratio in the range of 3–4 Gy for breast cancer (lower than the α/β ratio usually assumed for other tumours, i.e. 10 Gy), a large uncertainty affects both α and β parameter. To include in the study this variability, the TCP was calculated as a function of a wide range of α (β) for the fixed value of α/β.

Furthermore, considering that proliferation of the cell population is responsible for maintaining the tumor and that the primary target in the therapy of cancer is the clonogenic cell, the density of clonogens in a tumor is a key parameter in quantifying the response to therapy. Considering Figs. 5 and 6, large excursions in TCP can be observed for the implemented cell density over the range of α values considered. As expected, no matter the cell density, very steep TCP curves are obtained for increasing α values due to the assumption of Poisson distribution of surviving clonogens. Along these lines, at equal α values, the TCP is significantly lower for higher cell density. Taken together, Figs. 5 and 6 would seem to suggest that TCP will critically depend on the assumptions made about clonogenic cell density. Unfortunately, there are very few data on the variation of clonogenic cell density across tumours, both in vivo and in vitro. Despite the vast majority of tumor cells being eradicated after lumpectomy, cancer cell foci are likely to remain in the surgical bed, thus justifying treatment with post operative adjuvant therapies (mainly radiotherapy and radioablation) after surgery. Importantly, recent research indicates that the average cell density in breast microscopic disease volume is about 4.5 × 10^5^ cell/mm^3^ (standard deviation 8.4 × 10^4^ cells/mm^3^)^36^.This scenario is well represented by TCP curves reported in Figs. 5 and 6 (magenta and red curves). These curves have the potential to be used in the clinical practice right away for a given α value.

Moreover, the BAT-90 layer thickness could be not uniform, thus decreasing the uniformity of the activity distribution in the tumour bed plane and the dose along the z axis, ultimately affecting also the TCP. The radiobiological model is conservative, since, for a fixed ρ_0_, the N_s_ is overestimated. In fact, in the model, the residual clonogenic cells are uniformly distributed in the tumour bed plane, while the recommendation statement for pure DCIS includes resection margins negative at ≥ 3 mm. This means that the volume including residual cancer cells is overestimated in the model, resulting in an underestimation of the TCP values. In addition, the radiobiological model does not represent the inter- and intra-patient heterogeneity of the parameters; this usually results in less steep dose–response curves, as the TCP observed in the population is the average of the TCP values of patients with different radio-sensitivities.

The TCP was also described by means of a logistic dose response and the equations of isoeffect in the linear-quadratic model^37,38^. For D_50_ and γ parameters we used the values extrapolated in the analysis of the published START trail results performed by Giraudo et al*.*^24^.

This model is suitable for a treatment based on the BAT-90 device as the dose distribution is not uniform. In fact, for the evaluation of EUD we considered that homogeneous and heterogeneous absorbed dose distributions were equivalent from a radiobiological perspective if leading to the same fraction of surviving clonogens. Moreover, the calculation of the EUD values with respect to a RT schedule of 2 Gy per fraction, EUD_2Gy_, allows the comparison with other RT schedules and the application of the logistic model in Giraudo et al*.*^24^. As expected, in case of linearly decreasing cell density, the SF is lower than in case of constant density, therefore the TCP is higher.

The above considerations suggest that this approach is strongly supported in small lesions, similar to those identified by ASTRO/SSO; there is however room for improving the treatment of larger lesions, by further optimizing the dose as a function of the tumour bed radius. In addition, the treatment duration may play an important role in the optimization process. In fact, we are investigating new approaches which inject higher activity and fine-tune the treatment duration as a function of the planned dose. In this way it is possible to deliver a curative treatment in different clinical settings (i.e. tumour bed size) in an even shorter time, thus taking care of radiobiological and safety concerns. Moreover, the simulation setup adopted in the present study is well suited for the calculation of the dose in the target material, which is then used as the input in the calculation done using the radiobiological model. Anyway, for sake of completeness, we mention the possibility to use Geant4-DNA^39,40^, which provides electron tracking up to very low energy and distance scales and offers the possibility to implement radiobiological models, as an interesting future extension of this study.

Given the above considerations, the results of the present Monte Carlo simulation should be confirmed by prospective trials with clearly defined end-points and dosimetric assessments.

## Conclusion

A Monte Carlo simulation was used to calculate the activity needed to deliver a 20 Gy therapeutic isodose at the distance of interest from the tumour bed. The simulation includes several clinical and set-up issues: two bulks, composed of water or adipose tissue, were simulated and the delivered dose profile was estimated at different depths inside the two bulks. In addition, the effect of the BAT-90 layer thickness on the delivered dose profile was studied, showing a higher impact of a higher thickness on larger depths in both the simulated bulk tissues.

The radiobiological model developed in this study estimated the number of surviving cells, considering both a constant and a linearly decreasing clonogenic cell density. Different values for ρ_0_ were assumed, in order to take into account the heterogeneity of patient population and clinical scenarios. For the same reason, the TCP was estimated as a function of different radio-sensitivity values.

The results of both the Monte Carlo simulation and the theoretical radiobiological model show that BAT-90 represents a feasible therapeutic approach for the irradiation of surgical margins following resection of DCIS via BCS. The model predicts very solid results in case of small lesions, similar to those identified by ASTRO/SSO, while the activity/dose could be further optimized in case of larger lesions. Finally, a prospective clinical trial with defined end-points and dosimetric assessments is needed to validate the results of this model.

## Materials and methods

### BAT-90 medical device

The investigational device BAT-90 consists of a proprietary mix of two separate components, which are already individually CE-marked and FDA-approved as class III implantable medical devices: Bioglue^®^ (Cryolife Inc.) and SIR-Spheres^®^ (Sirtex Medical Ltd). The former is a bio-compatible mixture of Bovine Serum Albumin (BSA) (45%) and Glutaraldehyde (10%) in a 4:1 ratio. The latter (SIR-Spheres^®^) is a solution of biocompatible resin microspheres with diameter between 20 and 60 µm, coated with ^90^Y, a pure β^−^ emitter isotope with 64.1 h. half-life. The maximum and mean β particle energies emitted from ^90^Y are 2.26 MeV and 0.94 MeV, respectively, while the maximum and mean ranges of soft tissue penetration are 11 and 2.5 mm, respectively^41^. SIR-Spheres^®^ are currently indicated in unresectable liver cancers, both primary and metastatic, by intrahepatic intra-arterial administration (instruction for use: https://www.sirtex.com/media/169513/pi-ec-14-spheres-ifu-eu-row.pdf).

The ^90^Y-microsphere activity to be administered through BAT-90 is assessed using a personalised framework, taking into account the tumoricidal dose and the size of the target tissue to irradiate. BAT-90 is dispensed from a double-barrel syringe and mix within the delivery tip in a predefined ratio. The final mixed compound, i.e. the BAT-90 medical device, is delivered to the surgical room in a radio-protected box and administered homogeneously on the surgical cavity pushing on the pistons of the ^90^Y-loaded syringe, previously placed within a beta-shielding PMMA cylinder. The active compound takes a few seconds to reach full polymerization (30–120 s) resulting in an elastic BAT-90 layer adherent to the surgical bed. The entire absorbed dose is delivered to the tissue in about three weeks (about 91% of it in 9 days), after which the “exhausted” BAT-90 gets infiltrated by an inflammatory process, which completely replaces the matrix in about two years (resin microspheres are not further degraded).

### Monte Carlo simulation

The Geant4 simulation toolkit^21,42^ (version 10.5) was used to simulate the transport and interactions of the electrons emitted in the beta decay of ^90^Y, including the production of secondary particles and their subsequent interactions. The physics list G4EmStandardPhysics_option4^22^, which is implemented for the transport of low-energy electrons and photons, is adopted in this work with default production cuts. The full beta decay spectrum of ^90^Y has been simulated, as provided by the G4RadioactiveDecayPhysics package in Geant4.

**Figure 9:**
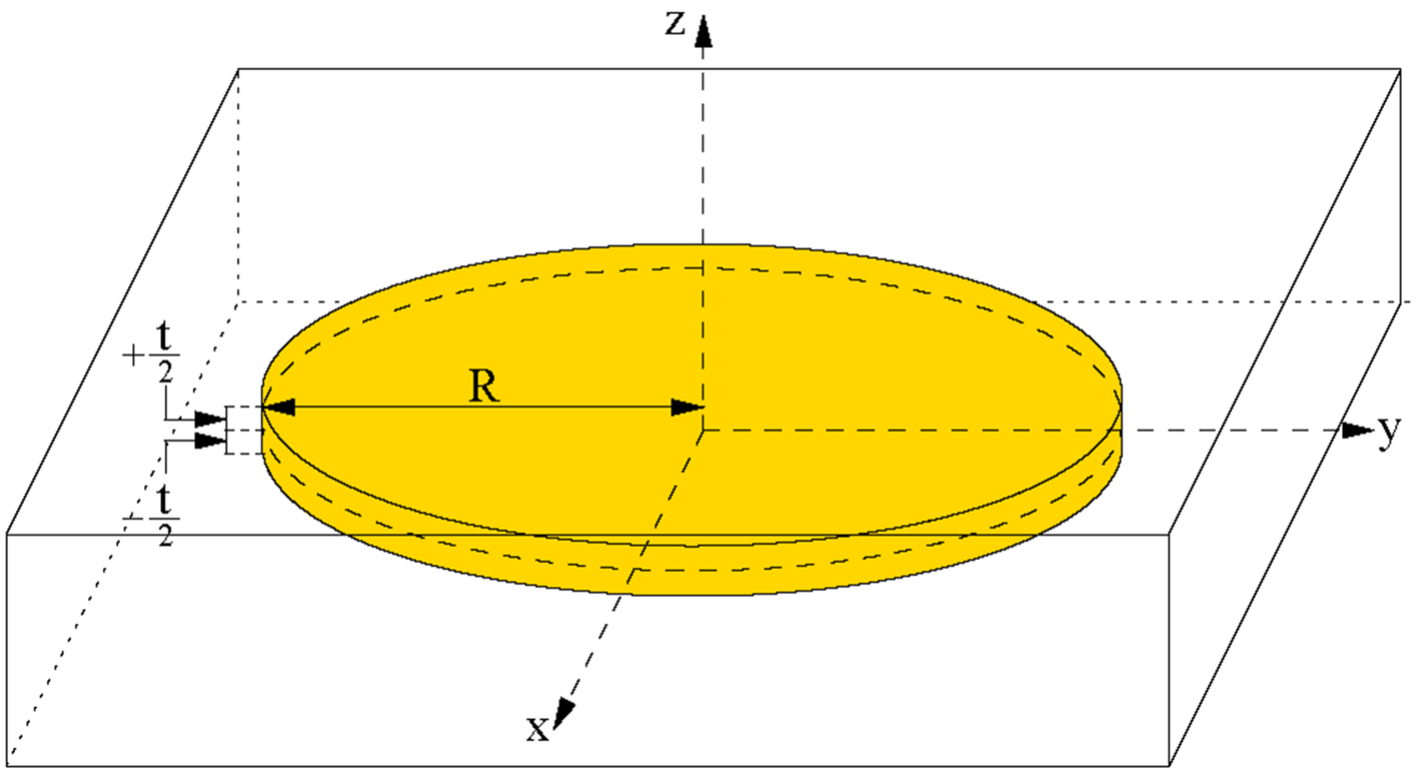
The simulation layout with the coordinate system definition. BAT-90 is represented by the yellow disk, whose center corresponds to the origin of the coordinate system.

A bulk of homogeneous material with a square section of 80 mm side and 25 mm thickness was segmented in cubic voxels of 1 mm side for the dose evaluation. A sketch of the simulation layout with the coordinate system definition is shown in Fig. 9.

In order to simulate BAT-90 deposition on the tumour bed plane, a homogenous ^90^Y layer of radius R is placed on a planar region in the middle of the bulk material. The tumour bed corresponds with the x–y plane of the coordinate system, while the z axis is normal to the tumour bed and the origin of the coordinates system is located in the center of the bulk material. In order to simplify the presentation of the results, a tumour bed of circular shape is assumed, centered at the origin of the reference system. Dose values are computed for each voxel, which are identified through the coordinates of their centers. Due to the geometrical symmetry of the simulation, the results are extracted using the cylindrical coordinates, z and the radial distance of the center of the voxel from the z axis r = √(x^2^ + y^2^).

Each simulated event consisted of one electron from the beta decay of ^90^Y, isotopically emitted from sources uniformly distributed inside the treated region.

It is reasonable to believe that in the clinical setting, the BAT-90 layer will have an inhomogeneous thickness over the whole tumour bed surface. In order to assess the impact of different BAT-90 layer thicknesses on the computed dose, a uniform distribution of ^90^Y nuclei in a cylindrical region with constant thickness t was assumed, with t values ranging from 0 to 5 mm.

The calculation was performed assuming water and adipose tissue as a bulk material (ICRP adipose tissue as implemented in the Geant4 toolkit: density = 0.95 g/cm^3^, element composition mass fractions: 11.4% H, 59.8% C, 0.7% N, 27.8% O, 0.1% Na, 0.1% Cl, 0.1% Ar).

The decay of 10^7^ primary ^90^Y nuclei was simulated for the dose calculation in each configuration. The activity corresponding to any given target dose was then obtained by assuming a complete decay of the radioactive nuclei (i.e. the BAT-90 remains in the tumour bed for an infinite time). For a treatment time much longer than the physical decay time of ^90^Y, the total number of decays N_dec_ depended on the injected activity A as follows:

1 $${N}_{dec}=\frac{A}{\lambda }$$

where λ is the decay constant. The dose profile calculated for 10^7^ decays was used to determine the activity needed to provide a 20 Gy isodose at a distance of interest from the tumour bed. The target volume is defined as the volume including the BAT-90 device and symmetric with respect to it, delimited by the 20 Gy isodose at a given distance z from the midplane of the BAT-90 layer. For illustration purposes, the results will be discussed for distances z = 0, 2, 4, 6, 8 mm and for thicknesses of the device t = 0, 1, 3, 5 mm.

### Radiobiological model

In order to implement the radiobiological model, the dose profile was fitted as a function of the distance z from the midplane of the BAT-90 layer by using the MATLAB software (R2017b, v.9.3.0):

2 $$d=a1*\mathrm{exp}(a2*\left(z-{z}_{0}\right))$$

where d is the normalized dose (Gy cm^2^/MBq) inside the tissues at the depth z-z_0_, where z_0_ = t/2 corresponds to the coordinate of the surface of the BAT-90 layer, and a1, a2 are the fit parameters selected by MATLAB with 95% confidence bounds.

For a tumour bed surface with area S, the delivered dose D is a function of the injected activity A and of z, and is:

3 $$D(z)=d(z) /S*A$$

namely, the total injected activity needed for the treatment is linearly dependent on the area of the tumour bed.

### Surviving fraction

For continuous therapy with a dose rate exponentially decreasing with time, the surviving fraction SF is given by^43,44^

4 $$S{F}_{z}=exp\left[-\alpha D(z)-\frac{\lambda \beta {D(z)}^{2}}{(\lambda +\mu )}\right]$$

where μ is the sublethal damage recovery constant^45^, β is the potential sparing capacity for a specific tissue or effect and α is the intrinsic radio-sensitivity.

If we consider an elemental target shell with tumour bed radius R, thickness dz at distance z from the midplane of the BAT-90 layer (Fig. 9), the volume is:

5 $${V}_{z}=S dz=\pi {R}^{2}dz$$

The number of cells still present in the elemental target volume after the surgery is

6 $$dN=\pi {R}^{2}\rho dz$$

where ρ is the residual clonogenic cell density.

Two different models were considered for clonogenic cell density: a constant density $\rho = {\rho }_{0}$ and a linearly decreasing density accordingly to $\rho ={\rho }_{0}\left(1-\frac{z-{z}_{0}}{k}\right)$.

The first model assumes that the residual tumour cells density is constant up to a distance equal to k from the surface of the Bat-90 layer and that the elemental target volume is totally composed of tumour cells.

The second model assumes that the tumour cell density decreases linearly as a function of the distance z = z_0_ + k, with ρ_0_ the residual clonogenic cell density at z = z_0_, and drops to zero in correspondence of the distance where the 20 Gy isodose is reached.

The total number of surviving cells in a shell with thickness equal to k, N_s_, is:

7 $${N}_{S}={\int }_{{z}_{0}}^{{z}_{0}+k}\pi {R}^{2}\rho (z)S{F}_{z}dz$$

The SF over the volume of interest is given by:

8 $$SF=\frac{{N}_{s}}{{N}_{0}}=\frac{{\int }_{{z}_{0}}^{{z}_{0}+k}\pi {R}^{2}\rho (z) S{F}_{z}dz}{{\int }_{{z}_{0}}^{{z}_{0}+k}\pi {R}^{2}\rho (z) dz}$$

where N_0_ is the number of clonogenic cells still present in the whole volume of interest after the surgery and before the irradiation.

### Tumor control probability

Tumor control probability models generally assume that the tumor is controlled if all the tumor clonogens are inactivated. Poisson statistics predict that the probability of this occurring is

9 $$TCP=\mathrm{exp}(-{N}_{S})$$

The model proposed by Niemierko et al.^37^, instead, assumed logistic dose response for the whole tumor and allows one to estimate the TCP when a tumor is irradiated nonuniformly:

10 $$TCP=\frac{1}{1+{\left(\frac{{D}_{50}}{EUD}\right)}^{4\gamma }}$$

where D_50_ is the total dose needed to obtain 50% of tumor control, γ is the normalized dose–response gradient calculated at D_50_ and EUD is the Equivalent uniform dose originally proposed by Niemierko et al.^33^. The EUD is defined as the dose that, if distributed uniformly in the target volume, will produce the same effect as the actual non-uniform dose distribution.

In this study, accordingly to previous works that estimated the D_50_ and γ parameters for breast tumour, the numerical value of the EUD was calculated with respect to a fractionation scheme for EBRT with 2 Gy fractions, i.e. EUD_2Gy_.

Based on this definition, the SF obtained with a non-uniform dose distribution can be expressed as:

11 $$SF=exp\left(-\alpha EU{D}_{2Gy}-\beta 2 EU{D}_{2Gy}\right)$$

For the dose delivery with the BAT-90 device, we first calculated the SF with Eq. (8) then we obtained the EUD_2Gy_ with Eq. (11).

### Comparison with other brachytherapy sources

To establish the benefit of the BAT-90 device, a comparison with other radiation sources used in partial breast irradiation was performed. In particular, the comparison focused on the Xoft Axxent electronic brachytherapy^46^ at different kVs, ^125^I seeds implants (Amersham 6702 source)^47,48^, and ^192^Ir HDR brachytherapy (MammoSite applicator)^49^.

The AAPM TG-43^48^ proposes a specific formalism in brachytherapy to evaluate the dose rate at point of coordinates $(r,\theta$):

12 $$\dot{D}(r, \theta )={S}_{K}\Lambda \frac{G\left(r, \theta \right)}{G\left({r}_{0}, {\theta }_{0}\right)}g\left(r\right)F(r, \theta )$$

where:

${\theta }_{0}=90^\circ$ and ${r}_{0}$ is a reference distance equal to 1 cm, ${S}_{K}$ is the air kerma strength, $\Lambda$ is the dose rate constant, $G\left(r, \theta \right)$ is the geometry factor, g(r) the radial dose function, and $F(r, \theta )$ is the anisotropy function. More details about these functions and factors can be found in AAPM TG-43^48^.

For the comparison, the function

13 $${f}_{X}=\frac{G\left(r, {\theta }_{0}\right)}{G\left({r}_{0}, {\theta }_{0}\right)} {g}_{X}(r)$$

was calculated, that represents the dose fall-off along the transverse axis of the source X considering the absorption, the scatter and also the geometrical distribution of radioactive material.

For the brachitherapy sources considered, the g_X_(r) for a point source approximation was assumed, with $G\left(r,\theta \right)={r}^{-2}$^46,48^, and $F(r, {\theta }_{0})$=1 for definition.

The dose profiles f_x_ for brachytherapy sources were then compared with the dose profile of BAT-90, f_BAT-90_, obtained normalizing d(z) in Eq. (2) to d(z = 1 cm). This profile, obtained with MC simulations, includes the effect of the scatter, attenuation and spatial distribution of radioactive sources of BAT-90 device.

## Supplementary Information

Supplementary Information.

## Data Availability

The data analysed in the current study are available from the corresponding author on reasonable request.

## Abbreviations

DCIS: Ductal carcinoma in situ
TCP: Tumour control probability
RT: Radiotherapy
BCS: Breast conserving surgery
LR: Local recurrence
APBI: Accelerated partial breast irradiation
EBRT: External beam radiation therapy
IORT: Intraoperative radiation therapy
PBI: Partial breast irradiation
SIRT: Selective internal radiotherapy
TARE: Transarterial radioembolization
WBI: Whole breast irradiation
IB: Intracavitary brachytherapy
MBI: Multi-catheter interstitial brachytherapy
LET: Linear energy transfer
RBE: Relative biologic effectiveness
BSA: Bovine serum albumin
SF: Surviving fraction
EUD: Equivalent uniform dose
