# Supplementary material for: Modelling a new approach for radio-ablation after resection of breast ductal carcinoma in-situ based on the BAT-90 medical device
Source: Sci Rep. 2022 Jan 7;12:14. doi: 10.1038/s41598-021-03807-6 (PMC8741759; doi:10.1038/s41598-021-03807-6)
Supplement: Supplementary file 1 — Supplementary Information. [file 41598_2021_3807_MOESM1_ESM.docx]

**Modelling a new approach for radio-ablation after resection of breast ductal carcinoma in-situ based on the BAT-90 medical device**

**Anna Sarnelli^1,*^, Matteo Negrini^2^, Emilio Mezzenga^1^, Giacomo Feliciani^1^, Marco D’Arienzo^3^, Antonino Amato^4^ and Giovanni Paganelli^5^**

^1^ Medical Physics Unit, IRCCS Istituto Romagnolo per lo Studio dei Tumori (IRST) “Dino Amadori”, Via P. Maroncelli 40, Meldola (FC), 47014, Italy

^2^ Istituto Nazionale di Fisica Nucleare, Sezione di Bologna, 40126 Bologna, Italy.

^3^ Medical Physics Unit, ASL Roma 6, Via Borgo Garibaldi 12, 00041 Albano Laziale RM, Italy

^4^ BetaGlue Technologies Spa, Lungadige Galtarossa 21, 37133 Verona, Italy

^5^ Nuclear Medicine Unit, IRCCS Istituto Romagnolo per lo Studio dei Tumori (IRST) “Dino Amadori”, Via P. Maroncelli 40, Meldola (FC), 47014, Italy

*** Corresponding author, anna.sarnelli@irst.emr.it**

Fig S1: TCP as a function of the R for a BAT-90 layer t=1.0 mm. A linearly decreasing residual clonogenic cells density is considered with different ρ_0_: 10^4^ cells/cm^3^ (magenta line) and 10^5^ cells/cm^3^ (red line)

Fig. S2: TCP as a function of the BAT-90 layer t for R= 30 mm. A linearly decreasing residual clonogenic cells density is considered with ρ_0_ = 10^4^ cells/cm^3^.
